# First Nighttime Primary Helicopter Emergency Medical Services (HEMS) Mission in Pediatric Severe Traumatic Brain Injury: Establishing a Functional Night Landing Network in a Resource-Limited Setting

**DOI:** 10.7759/cureus.109080

**Published:** 2026-05-18

**Authors:** Orazio Stefano Giovanni Filippelli, Nicola Polimeni, Carmelo Romano

**Affiliations:** 1 Department of Anesthesia and Intensive Care, Azienda Ospedaliero Universitaria Renato Dulbecco, Catanzaro, ITA; 2 Department of Anesthesia and Intensive Care, Grande Ospedale Metropolitano, Reggio Calabria, ITA; 3 Department of Anesthesia and Critical Care, Azienda Ospedaliero Universitaria Renato Dulbecco, Catanzaro, ITA

**Keywords:** helicopter emergency medical service, nighttime hems, pediatric trauma, pediatric traumatic brain injury, prehospital critical care, rapid sequence intubation (rsi)

## Abstract

Severe traumatic brain injury (TBI) in pediatric patients is a time-dependent condition in which early airway management and rapid access to neurosurgical care are considered important to reduce the risk of secondary brain injury. The potential utility of Helicopter Emergency Medical Services (HEMS) is closely related to the availability of adequate landing sites, particularly during nighttime operations. In Calabria (Southern Italy), nighttime HEMS activity has historically been limited by a marked lack of certified hospital helipads, especially within tertiary (hub) centers. We report the case of a pediatric patient involved in a bicycle-versus-car collision, presenting with severe TBI (Glasgow Coma Scale (GCS) score of 6), managed during one of the first nighttime primary HEMS missions performed after the recent regional implementation of alternative illuminated landing sites. Due to the absence of hospital-based nighttime landing facilities, a rendezvous point was performed at a municipal sports field, 1 of approximately 20 sites recently identified and equipped across the region to support nighttime operations. On HEMS arrival, the patient exhibited impaired consciousness and inadequate airway protection. Rapid sequence intubation (RSI) was performed on scene, followed by helicopter transport to a tertiary neurosurgical center, where urgent surgical evacuation of an epidural hematoma was carried out. The patient showed favorable neurological recovery, with no clinically evident major focal neurological deficits after a three-day postoperative intensive care stay. This case highlights the operational feasibility and potential clinical value of nighttime primary HEMS missions in a structurally constrained environment. The implementation of a distributed network of alternative landing sites, mainly based on sports facilities, may represent a preliminary operational step toward a more functional regional H24 HEMS system, partially compensating for the lack of dedicated hospital helipads. Early advanced airway management combined with rapid transfer to neurosurgical care may have contributed to the favorable outcome in this patient; however, the clinical evolution was likely influenced by multiple factors, including the presence of a surgically treatable epidural hematoma and timely neurosurgical evacuation.

## Introduction

Severe traumatic brain injury (TBI) is one of the leading causes of mortality and long-term neurological disability in pediatric populations. The early phase of management is crucial, as patient outcomes are strongly influenced by secondary brain injury, which is primarily driven by hypoxia, hypercapnia, hypotension, and impaired cerebral perfusion [1,2].

Among these factors, hypoxia is one of the most significant predictors of poor outcome. Even brief episodes of oxygen desaturation can lead to substantial worsening of neurological prognosis. For this reason, early and advanced airway management represents a cornerstone in the treatment of severe TBI. Current international guidelines support endotracheal intubation in patients with severe traumatic brain injury and a Glasgow Coma Scale (GCS) of ≤8, particularly when protective airway reflexes are compromised [1]. In pediatric severe TBI, airway management should be performed while minimizing secondary insults such as hypoxia, hypotension, and inappropriate ventilation.

In this context, prehospital rapid sequence intubation (RSI), when performed by trained physicians [3], allows rapid airway control with the aim of improving oxygenation and ventilation while reducing the risk of aspiration and secondary brain injury.

At the same time, Helicopter Emergency Medical Services (HEMS) have become an essential component of modern emergency systems [4-6], providing rapid access to the scene, advanced prehospital medical care, and expedited transport to specialized referral centers.

However, the potential utility of HEMS is highly dependent on infrastructure availability, especially for nighttime operations. The availability of certified hospital helipads is crucial to guarantee continuous 24-hour (H24) service.

In the context of the Calabria region, the HEMS system has historically been affected by significant structural limitations. At the time of the case described, nighttime-capable hospital helipads were extremely limited and, notably, completely absent in all three regional hub hospitals. Only a small number of spoke hospitals, such as Locri and Polistena, were equipped for nighttime helicopter operations.

This configuration resulted in a markedly restricted operational network, with potential delays in access to definitive care for time-sensitive conditions such as severe TBI.

To address these limitations, a recent regional reorganization introduced a network of alternative nighttime landing sites, mainly consisting of municipal sports facilities distributed across the territory (approximately 20), specifically identified and equipped to enable safe nighttime HEMS operations.

The case presented here occurred within this transitional organizational framework and represents one of the first documented primary nighttime HEMS missions performed after the regional implementation of alternative nighttime landing sites, thus constituting an event of both clinical and system-level relevance.

This report aims to describe the clinical case and discuss the role of early advanced management and air transport within a regional emergency system characterized by significant infrastructural constraints and progressive development of a more integrated H24 HEMS network.

## Case presentation

The event occurred during nighttime hours in August 2025 and led to the activation of the regional HEMS as a primary mission. The patient was a male pediatric trauma victim involved in a bicycle-versus-car collision in the Roccella Ionica area, Calabria, Southern Italy. According to the prehospital reconstruction, the child, while riding a bicycle, collided with a motor vehicle and sustained a major head injury, followed by marked impairment of consciousness.

Initial management was provided by a ground emergency medical team without an onboard critical care physician, which transported the patient from the scene to the rendezvous point. The accident occurred in the mountainous area of Nardodipace, and the ground transfer to Roccella Ionica covered a considerable distance (approximately 30-35 km) along secondary provincial roads characterized by low average speed and multiple curves, resulting in prolonged transport time despite the relatively short distance.

This mission had particular operational relevance because it represented one of the first documented nighttime primary HEMS interventions performed after the recent regional implementation of alternative nighttime landing sites. Due to the absence of nighttime hospital helipads at hub centers and the need to reach the patient rapidly in the field, a rendezvous point was established at the municipal sports field in Roccella Ionica, one of the recently identified sites suitable for nighttime landing operations. The site had been previously identified for nighttime HEMS operations and included dedicated lighting, ground vehicle accessibility, safety perimeter preparation, and coordinated communication between ground EMS personnel and the HEMS crew.

The HEMS crew departed from the Lamezia Terme airport base and reached the rendezvous site. Shortly after the helicopter’s arrival, the ground ambulance from Nardodipace also arrived, carrying the patient. The patient was therefore managed within a complex operational scenario involving a nighttime mission, a non-hospital landing site, severe pediatric trauma, and the need for rapid stabilization before transport.

On initial assessment by the HEMS team, the patient, an 11-year-old male with normal body habitus for age, showed severe neurological impairment, with a GCS score of 6, consistent with severe traumatic brain injury. The level of consciousness was markedly reduced, and the patient was unable to adequately protect the airway, exposing him to a high risk of hypoxia, aspiration, and progression of secondary brain injury. Pupils were equal, round, and reactive to light. Hemodynamic parameters were preserved, with a documented blood pressure of 139/90 mmHg and a heart rate of approximately 89 bpm, associated with maintained peripheral perfusion and no signs of circulatory instability. Oxygen administration had already been initiated during ground transport, and cervical spine precautions were maintained throughout prehospital management. Clinical examination revealed major cranial trauma without immediately evident signs of severe extracranial injury. Neurological impairment, therefore, represented the primary clinical concern at presentation. Given the GCS score of 6, impaired consciousness, and inability to ensure airway protection, rapid sequence intubation was indicated [1,3]. Pharmacological induction was performed using intravenous midazolam (5 mg), rocuronium (20 mg), and atropine (0.5 mg), followed by successful first-pass endotracheal intubation with a 5.5-mm cuffed endotracheal tube. Correct tube placement was confirmed clinically. Pre-intubation peripheral oxygen saturation was 98%. Peripheral oxygen saturation remained stable during transport with assisted ventilation and supplemental oxygen administration using a pediatric self-inflating bag. Blood pressure remained preserved, with no clinically evident peri-intubation hypotension. No peri-intubation complications, including clinically evident hypoxemia or hemodynamic instability, were observed. Ventilation during helicopter and subsequent ground transport was manually maintained using a pediatric self-inflating bag. Post-intubation sedation and neuromuscular blockade were maintained with repeated boluses of midazolam and rocuronium according to standard pediatric practice. Continuous End-tidal carbon dioxide (EtCO_2_) values were not available in the retrospective prehospital documentation. The airway management strategy was aimed at supporting adequate oxygenation and ventilation during transport while reducing the risk of aspiration and secondary neurological deterioration.

After intubation, the patient was stabilized and prepared for air transport. The decision to transfer the patient to a neurosurgical hub was based on the severity of the head injury, the low GCS score, and the high suspicion of clinically significant intracranial injury requiring neurosurgical evaluation. The overall transport process required multiple sequential transfers, including ground transport from the scene to the rendezvous point, helicopter transfer to Reggio Calabria airport, and subsequent ground transport by ambulance to the tertiary hospital. This multimodal pathway reflects the current regional logistical configuration. Because this report focuses on time-dependent trauma care and nighttime HEMS logistics, a detailed operational timeline of the prehospital and in-hospital management is provided in Table 1.

**Table 1:** Chronological timeline of prehospital, transport, and in-hospital management Chronological sequence of prehospital stabilization, multimodal transport, neuroimaging, neurosurgical intervention, and early postoperative management in a pediatric patient with severe traumatic brain injury requiring nighttime primary Helicopter Emergency Medical Services (HEMS) activation. Approximate times are indicated where exact timestamps were unavailable from retrospective clinical and operational documentation.

Phase	Event	Time
Prehospital phase	Estimated time of injury	~20:00
Prehospital phase	HEMS activation	20:25
Air mission	HEMS takeoff	20:35
Rendezvous operations	HEMS arrival at rendezvous point	21:09
Rendezvous operations	Ground EMS arrival at rendezvous point	21:10
Critical care intervention	Rapid sequence intubation (RSI)	21:20
Transport phase	Departure from rendezvous point	21:36
Air transport	Arrival at Reggio Calabria airport	22:08
Secondary ground transfer	Arrival at neurosurgical center emergency department	22:20
In-hospital evaluation	CT acquisition	22:35
Neurosurgical management	Emergency craniotomy	00:10
Postoperative course	Extubation	Postoperative day 2
Postoperative course	ICU stay	3 days
Clinical outcome	Transfer to pediatric ward	Postoperative day 3
Clinical outcome	Neurological status at transfer	Awake, cooperative, spontaneous breathing, without clinically evident major focal neurological deficits

The patient was then transported by helicopter to the Grande Ospedale Metropolitano of Reggio Calabria, a tertiary referral center with neurosurgical capability.

Upon hospital arrival, the patient underwent urgent diagnostic evaluation. Initial head CT revealed a large acute left temporo-parieto-occipital epidural hematoma measuring up to 22 mm in thickness, associated with diffuse cerebral edema, near-complete effacement of cortical sulci, compression of the left lateral ventricle, and approximately 9 mm rightward midline shift. A left temporal bone fracture extending toward the ipsilateral mastoid region was also identified (Figure 1). Subsequent hospital evaluation also documented anisocoria associated with the progression of intracranial mass effect.

**Figure 1:**
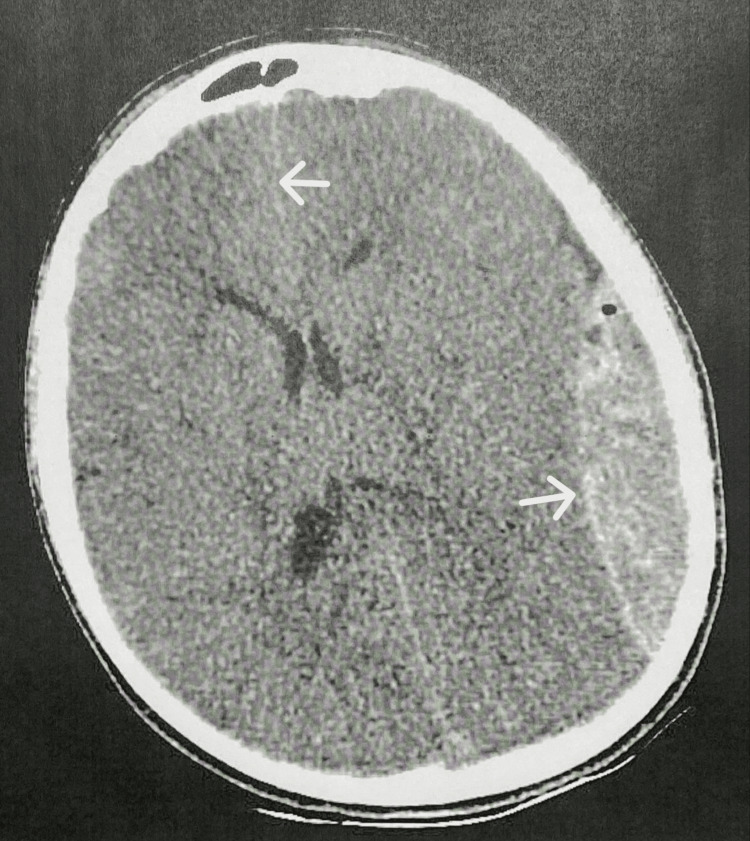
Axial head CT showing epidural hematoma and midline shift Axial head CT scan demonstrating a large left temporo-parieto-occipital epidural hematoma (white arrow), associated with diffuse cerebral edema, marked mass effect with rightward midline shift (white arrowhead), sulcal effacement, and frontal pneumocephalus.

Emergency neurosurgical intervention was therefore performed. Surgical management consisted of a left fronto-temporo-parietal craniotomy with evacuation of the epidural hematoma, hemostasis, and repair of the associated skull fracture. Intraoperatively, a large epidural blood clot under pressure was identified and carefully evacuated. A bleeding meningeal arterial branch was coagulated, and the bone flap was subsequently repositioned and fixed after hemostatic control. No decompressive craniectomy was required. Intracranial pressure monitoring was not used during postoperative intensive care management.

The interval from estimated injury to CT acquisition was approximately 2 hours and 35 minutes, while the interval between CT diagnosis and initiation of urgent neurosurgical intervention was approximately 1 hour and 35 minutes. The patient subsequently underwent postoperative intensive care management with progressive neurological improvement. He was successfully extubated on postoperative day 2 and later transferred to the pediatric ward awake, cooperative, breathing spontaneously in room air, and without clinically evident major focal neurological deficits.

This case illustrates the complexity of time-dependent pediatric trauma management within a nighttime multimodal HEMS-supported transfer pathway in a structurally constrained regional emergency system.

## Discussion

The present case has a dual relevance. On one hand, it describes the time-dependent clinical management of a pediatric patient with severe traumatic brain injury; on the other, it highlights the organizational value of a nighttime primary HEMS mission performed in a regional setting characterized by significant infrastructural limitations and an emergency network undergoing progressive development.

In severe traumatic brain injury, particularly in pediatric patients, the prehospital phase is crucial. Primary brain injury is directly related to the traumatic event, but prognosis is strongly influenced by secondary brain injury, which may develop in the minutes and hours following trauma. Hypoxia, hypercapnia, hypotension, inadequate ventilation, and delayed access to neurosurgical care may all contribute to worse neurological outcomes [1,2,7].

In the present patient, the dominant clinical finding on HEMS arrival was severe neurological impairment, with a GCS score of 6. This finding identifies severe traumatic brain injury and, in the presence of impaired consciousness and inability to protect the airway, represents a clear indication for advanced airway management. The decision to perform rapid sequence intubation on scene [3] was therefore aimed at supporting adequate oxygenation and ventilation during transport while reducing the risk of aspiration and secondary neurological deterioration. However, in severe traumatic brain injury, the potential benefit of prehospital RSI is closely dependent on procedural quality and physiological management, as peri-intubation hypoxia, hypotension, and inappropriate ventilation may worsen secondary brain injury [1,3].

This point is central to the case. Prehospital care was not limited to rapid transport alone; rather, it included an advanced, time-critical clinical intervention. In pediatric severe traumatic brain injury, even brief episodes of hypoxia may have relevant neurological consequences [8]. Early intubation before transport allowed airway stabilization and may have reduced the risk of secondary brain injury during a logistically complex mission.

The second relevant element is the role of HEMS. In major trauma, HEMS combines two major components [4-6]: the availability of advanced clinical expertise directly at the scene and the possibility of rapid transfer to specialized centers. In this case, transport to the Grande Ospedale Metropolitano of Reggio Calabria provided access to a neurosurgical center, where urgent evacuation of a large epidural hematoma was performed. The favorable subsequent course cannot be attributed to a single factor, but appears consistent with a coordinated chain of care that included preserved hemodynamic status, recognition of severity, advanced airway management, stabilization, rapid transport, the presence of a surgically treatable epidural hematoma, and timely neurosurgical evacuation.

However, the most original aspect of this case lies in its operational context. The mission described represents one of the first documented nighttime primary HEMS interventions performed after the recent regional implementation of alternative nighttime landing sites. In Calabria, at the time of the case, nighttime hospital landing sites were available only in a very small number of spoke hospitals, while none of the three regional hub hospitals were equipped with certified in-hospital helipads for nighttime HEMS operations. This has major organizational implications, as it limits the full integration of HEMS into time-dependent emergency networks, especially during nighttime hours. Importantly, the main infrastructural limitation involved both the scarcity of nighttime-capable territorial landing sites and the absence of certified nighttime hospital helipads at regional hub centers. In the present case, the newly implemented sports-field landing network enabled nighttime rendezvous point and primary HEMS activation in the field; however, a secondary ground transfer to the neurosurgical hospital remained necessary after helicopter transport.

The recent identification of approximately 20 sports facilities distributed across the regional territory as nighttime landing sites may represent an important operational development for the regional HEMS system. These sites, introduced operationally only a few weeks before the present case during a regional reorganization in mid-2025, do not fully replace dedicated hospital helipads, but provide an intermediate operational solution capable of extending nighttime HEMS coverage [9]. The rendezvous performed at the sports field in Roccella Ionica suggests how this alternative network may make primary nighttime missions feasible even in areas lacking dedicated hospital infrastructure. Historically, the capacity for primary nighttime HEMS missions in the region had remained markedly limited for decades, reflecting a long-standing organizational gap that has only recently begun to be addressed.

The geographical characteristics of the region further emphasize this limitation. The patient was initially transported from a mountainous inland area (Nardodipace) to the coastal rendezvous point in Roccella Ionica, covering a considerable distance along secondary provincial roads characterized by low average travel speed and multiple curves. This prolonged ground transport phase, combined with the need for subsequent air and ground transfers, reflects the complexity of the current regional emergency logistics. The overall interval from estimated injury to arrival at the neurosurgical center was approximately 2 hours and 20 minutes, including scene management, rendezvous operations, prehospital RSI, and multimodal transport. Considering the mountainous inland location of the accident, the characteristics of the regional road network, and nighttime operating conditions, direct ground transport to the neurosurgical referral center would likely have required substantially longer transfer times.

This represents the key system-level message of the case: an incomplete system may still support feasible and potentially beneficial care when supported by territorial planning, coordination between ground and air emergency services, advanced medical expertise, and appropriate patient selection. The objective timeline presented in Table 1 suggests that the contribution of HEMS in this case was not limited to transport alone. The HEMS system also enabled earlier physician-level critical care interventions, including rapid sequence intubation and advanced airway stabilization, before definitive neurosurgical treatment. In the described regional setting, physician-staffed prehospital critical care resources with specific expertise in advanced pediatric airway management were limited within the regional ground EMS system. In this context, the sports-field landing site functioned as an operational component of the regional emergency network. In the absence of nighttime helipads at hub hospitals, the use of alternative landing sites may have facilitated earlier deployment of the advanced team to the patient, allowing critical interventions such as intubation and rapid transfer to a specialized center. In this case, the patient underwent multiple sequential transfers, including ground transport from the scene to the rendezvous point, helicopter transfer to the nearest airport, and subsequent ground transport to the tertiary hospital. Such a multimodal pathway highlights the current structural complexity of the system. At the same time, indirect transfer pathways involving multiple sequential transfers may also introduce potential delays, logistical complexity, and additional clinical risks compared with direct transport to hospitals equipped with certified nighttime helipads.

The pediatric nature of the case further increases its relevance [10]. Severe traumatic brain injury in children requires a particularly rapid and competent response, as physiological reserve may be limited and the margin for error reduced. Pediatric airway management, pharmacological induction, ventilation, and safe transport require specific expertise and team coordination. In this sense, the availability of a HEMS team capable of performing RSI in the field may represent a relevant operational advantage compared with a model based solely on transport, particularly when supported by appropriate training, procedural governance, physiological monitoring, and adequate post-intubation management.

This case also highlights an important methodological point: the operational role of HEMS should not be assessed only in terms of flight time or distance covered [6], but also in terms of the system’s ability to bring advanced clinical competence to the patient at the right time. In pediatric severe traumatic brain injury, the potential benefit is not merely reaching the hospital earlier, but reducing the risk of hypoxia, aspiration, inadequate ventilation, and delayed decision-making during the prehospital phase. From this perspective, the potential value of HEMS in the present case was related not only to transport but also to the early deployment of a physician-led critical care team capable of advanced airway management and stabilization within a complex multimodal transfer pathway.

This case should therefore not be interpreted merely as an isolated clinical success, but as an early demonstration of system feasibility in a transitioning emergency network. The mission described may therefore be interpreted as a sentinel case illustrating early operational feasibility within a transitioning regional nighttime HEMS network. It does not, by itself, prove the overall effectiveness of a regional nighttime HEMS network, but it suggests that the activation of alternative landing sites may represent a concrete first step toward a more functional and integrated H24 emergency network. The favorable outcome observed in this patient may support the clinical plausibility of this organizational approach. However, the observation should be considered hypothesis-generating and interpreted cautiously, as this is a single case report.

Nevertheless, several critical issues remain. A network based on sports facilities requires clear operational standards, regular maintenance, adequate nighttime lighting and visibility, safe accessibility for ground vehicles, weather-related operational assessment, shared procedures, reliable communication between dispatch centers, ground EMS teams, and HEMS crews, as well as continuous personnel training. In addition, these alternative landing sites require clearly defined medico-legal responsibilities and continuous quality monitoring to ensure safe integration within the regional emergency network. Furthermore, the absence of nighttime helipads in the main hub hospitals remains a significant limitation, as it requires indirect transfer solutions that may be less efficient and may expose patients to additional logistical complexity and transfer-related risks. The use of sports fields should therefore be considered an evolutionary step rather than a definitive endpoint.

In conclusion, this case shows how the combination of advanced clinical decision-making and organizational adaptation may produce a favorable outcome even in a complex infrastructural context. This nighttime primary HEMS mission, performed after the recent regional implementation of alternative nighttime landing sites and applied to pediatric severe traumatic brain injury, therefore has a value that extends beyond the individual event: it suggests the possibility of progressively building a more functional and integrated H24 emergency network, capable of responding to the most critical time-dependent conditions. This evolution will require not only infrastructural development but also continued integration between clinical expertise and territorial organization.

## Conclusions

The present case suggests that, in pediatric severe traumatic brain injury, timely and appropriate prehospital management may contribute to favorable clinical outcomes. In particular, early airway control through rapid sequence intubation, combined with prompt transfer to a neurosurgical referral center, may represent an important component of early neuroprotective management. Beyond its clinical relevance, this case carries significant organizational implications. It represents one of the first documented nighttime primary HEMS missions performed after the recent regional implementation of alternative nighttime landing sites within a regional system characterized by major infrastructural limitations, particularly regarding the lack of certified hospital helipads for nighttime operations in hub centers. In this context, the use of alternative landing sites, mainly identified in sports facilities distributed across the territory, allowed partial mitigation of logistical constraints and enabled timely advanced intervention. This experience suggests that the implementation of a distributed network of nighttime landing sites, even outside hospital settings, may represent a pragmatic operational solution to extend HEMS operational capacity during nighttime hours, especially in regions where dedicated infrastructure is lacking. Although such an approach cannot replace the need for certified hospital helipads at referral centers, it may constitute a concrete step toward the development of a more functional 24-hour HEMS network. Within this framework, the case may be interpreted as a sentinel case, suggesting how the integration of advanced clinical expertise, territorial organization, and operational adaptability may contribute to favorable outcomes even in structurally constrained environments. The combination of timely clinical decision-making and system-level flexibility may represent an important factor in the management of time-dependent critical conditions. Further studies and systematic observations are required to assess the broader impact of such organizational models. Nevertheless, the present case suggests that the progressive development of nighttime HEMS networks, through the integration of alternative landing sites and gradual infrastructural enhancement, may contribute to improving the management of pediatric severe traumatic brain injury and other time-dependent emergencies in structurally constrained regions, although further systematic evaluation is required.
